# Esophageal disorders in mixed connective tissue diseases

**Published:** 2016

**Authors:** AE Nica, LM Alexa, AO Ionescu, O Andronic, DN Păduraru

**Affiliations:** * Department of Anesthesiology, University Emergency Hospital Bucharest, Romania; ** ”Carol Davila” University of Medicine and Pharmacy, Bucharest, Romania; *** Neurorehabilitation Clinic, Rehabilitation Hospital, Iași, Romania; **** III rd Department of General Surgery, University Emergency Hospital Bucharest, Romania

**Keywords:** mixed connective tissue disease, esophageal manifestations, digestive disorders

## Abstract

Extra Musculoskeletal manifestations are a distinct clinical entity that refers to a combination of clinical features, which are found in multiple rheumatic diseases. Besides the standard manifestations, other organs can be damaged such as the vascular system, skin, gastrointestinal tract, musculoskeletal system, cardiopulmonary system, hematologic system, kidneys, and the central nervous system. Among the gastrointestinal MCTD symptoms, the most frequent are the esophageal ones. Treatment of patients with MCTD must be performed by both medical and surgical multidisciplinary teams in order to provide a management suitable for the patients’ needs.

All authors have contributed significantly and have been involved in the writing of the manuscript in draft and any revision stages, and have read and approved its final version.

## Introduction

Extra Musculoskeletal manifestations represent a distinct clinical entity, which acts similar to a combination of clinical features, found in multiple rheumatic diseases.

Mixed connective tissue disease (MCTD), the old name for Sharp’s syndrome, was first described in 1972 as a connective tissue disorder with common particularities also found in the Systemic Lupus Erythematosus (SLE), the Systemic Sclerosis (SS) and the Polymyositis (PM). In the past, a high titer of Autoantibodies (anti-U1-RNP) were specific to MCTD, but later, a high prevalence of arthritis which resembled rheumatoid arthritis (RA) was observed in patients with MCTD [**1**,**2**].

Frequently, the clinical characteristics of MCTD take place after a few years, so the full clinical picture is rarely present from the start. In the early stages, patients often exhibit one of the following characteristics: Raynaud’s phenomenon, swelling of the hands (puffy fingers), sclerodactyly, arthralgia, arthritis, myalgia, myositis, or impaired general condition. These are most often accompanied by pulmonary condition and esophageal symptoms [**3**]. 

Until this moment, it was not possible to conceive a universally accepted diagnostic criterion. There are four different types of criteria: the 1987 Sharp’s criteria, the 1987 Alarcon-Segovia, the 1987 and 1991 Kahn Kasukawa, but none of the aforementioned criteria is considered superior, leading to a simultaneous usage. Positive diagnosis of certainty requires the presence of Antibodies Anti-U1-RNP [**4**].

Besides the classic manifestations, almost any organ can be impaired: the vascular system, skin, gastrointestinal tract, musculoskeletal system, cardiopulmonary system, hematologic system, kidneys, and the central nervous system [**5**].

**Esophageal manifestations in MCTD**

Amongst the gastrointestinal manifestations of the MCTD, the esophageal symptoms were the most common, being found in about 85% of the cases. These disorders appeared both in the upper third of the striated muscles and the lower ⅔, containing smooth muscle [**6**].

There is still unclear why MCTD causes esophageal complications and there are not many studies on this subject. One such study is the work led by Akihisa Kamataki, which investigated 27 cases of postmortem patients with MCTD. Out of the 27 cases, 25 had histopathological changes in the esophagus. All the changes observed were located in the lower ⅔ of the esophagus. Regarding the muscle layers, the circular layer was affected largely than the longitudinal layer, most cases without identifying greater longitudinal lesions. The changes observed were reflected in the severe atrophy with a lack of muscle fiber in some places, up to the fibrosis of the muscular layer [**2**].

In some studies, the esophageal dysfunction was associated with extracellular matrix degradation, vascular disorders and AutoAntibodies, without the pathophysiological mechanism being fully explained [**7**].

The esophageal dysmotility occurs in 45% to 85% of the patients and is frequently subclinical at the onset of MCTD. Like the SS, the esophageal manometry and barium swallow may show a reduction in peristalsis, especially in the lower third and low pressure of the lower esophageal sphincter. The gastroesophageal reflux and the swallowing problems may occur as secondary events in advanced stages. These manifestations are as frequent as in SS, but less upsetting [**5**].

Schneider et al. conducted a study on a batch of 39 patients and examined the gullet effects of MCTD in comparison to other pathologies with the help of esophageal manometry. Although they were statistically questionable, the results of the study revealed that the MCTD does not induce specific symptoms compared to other pathologies. This is very important because MCTD should be considered as a differential diagnosis in patients with different esophageal pathologies. 14 patients had connective tissue diseases and 25 of them had chest pain, without tissue disorders. With the help of esophageal manometry, changes of motility were recorded such as aperistalsis (lack of peristaltism) in the lower ⅔, with decreasing pressure of the lower esophageal sphincter, and one case associated upper sphincter pressure drop. Changes in the upper third may have multiple other causes such as muscular dystrophy, myasthenia gravis, and motor neuron disease. Typically, two-thirds of the lower anomalies occur due to non-rheumatic diseases (Multiple Sclerosis, Diabetes Mellitus, Hypothyroidism), as confirmed by the study undergone by Schneider [**8**].

The esophageal features under this condition may be the cause of pulmonary symptoms such as cough, asthma, or bronchitis. Recently, it has been questioned whether there is any connection between the gastroesophageal reflux and the pulmonary fibrosis in MCTD. The question is justified given that there are no studies that prove a link between the gastroesophageal reflux and the idiopathic pulmonary fibrosis.

Following Kasukawa classification criteria, 50 patients with MCTD were included in a prospective study conducted by the Department of Rheumatology in the Sao Paulo Clinical Hospital during 2001 and 2003. 39 of 50 patients showed parenchymal abnormalities on HRCT and 28 had an esophageal dilatation. Further, 18 of 36 patients had gastroesophageal reflux and 30 of 36 had an esophageal motor dysfunction. Interstitial syndrome incidence was significantly higher in patients with esophageal dilatation (92% vs. 45%; p < 0.01) and in patients with severe motor dysfunction (90% vs. 35%; p < 0.001). Aperistaltic patients had lower values of carbon monoxide diffusing capacity than those with normal gullet or those with moderate dysfunction of the esophagus (76.4% vs. 96.6%) without recording appreciable differences in the total lung capacity (81% to 93%) [**9**].

Dyspnea is more commonly identified in patients with esophageal aperistalsis and with moderately affected peristalsis, being present in over 70% of the cases, compared to those with a normal motor function of whom only 20% had difficulty breathing. The interstitial pulmonary disease was identified radiographically as matt glass opacities type in 85% of the aperistaltic patients [**10**-**14**].

It is difficult to prove a causal relationship between the impaired esophageal function and the pulmonary dysfunction, the results still being questionable. While Marie’s study revealed a correlation between the severity of the esophageal motor damage and the interstitial pulmonary syndrome, confirming the importance of acid reflux in determining the lung disease [**10**], the study undertaken by Troshinski failed to demonstrate the same results. Further, Caleiro [**15**] failed to emphasize the relationship between impaired interstitial lung and esophageal dysfunction by means of scintigraphy, and could not prove a cause and effect connection in patients with MCTD [**11**]. However, the research revealed a link between the reflux of food and the pulmonary implications because chronic aspiration induces centrolobular fibrosis. Thus, in the absence of acid reflux, aperistalsis determines interstitial syndrome. Food reflux is involved in interstitial disease, and in the context of MCTD, it has an impact on patients with esophageal dilatation revealed on CT scans and aperistalsis shown in esophageal manometry [**3**,**16**-**18**].

Besides the esophageal damage, peristalsis disturbance can also occur in other segments of the gastrointestinal tract. Among these, others can be enclosed, as for instance, delayed gastric emptying (6% of the patients), bowel transit disorders like slow intestinal transit and dilatations. Malabsorption can occur due to bacterial growth and the expansion of the small intestine. Pseudodiverticula and colonic perforations were also reported with an increased frequency in patients with MCTD [**19**-**20**].

The esophageal component of MCTD may present some complications during the course of the disease, among which:

• chronic reflux esophagitis

• Candida esophagitis by infection due to the incomplete emptying of the esophagus, acid reflux, and cortisone therapy 

• esophageal stenosis 

• upper gastrointestinal hemorrhage

• Barrett metaplasia with risk of developing adenocarcinoma 

**Treatment of gastrointestinal disorders**

Gastroesophageal reflux should be treated with proton pump inhibitors (PPI). Patients with severe esophageal dysfunction may benefit from corticosteroid therapy, which increases pressure of cardia and improves esophageal peristalsis. Doctors at Sheba Medical Center in Tel Hashomer, Israel, displayed a case of clinical and radiological remission of esophageal disorders following the treatment with Fluocortolone in a patient with MCTD. Although corticosteroids have not proved effective during the progression of systemic sclerosis, doctors in Israel demonstrated that in the early stages of inflammatory MCTD, patients receiving corticosteroids have a superior evolution compared to the patients who did not follow the therapy or those who did not have the therapy [**6**,**9**,**21**,**22**].

Complications such as dysphagia secondary to strictures/ stenosis or mucosal rings should not occur if the treatment is performed regularly with PPI, but, if complications occur, the use of endoscopic expansions might be required. Screening for Barrett’s esophagus should be indicated in patients with severe or long-term esophageal reflux [**23**].

## Conclusions

The clinician must consider the rheumatic etiology in the diagnosis of patients with digestive symptoms and, in particular, esophageal pathology. Also, the extra musculoskeletal manifestations of patients with mixed connective tissue disease can seriously affect their quality of life, therefore it is essential to be investigated and discussed in both surgical and medical multidisciplinary teams, in order to provide the suitable management treatment.

All the authors have contributed significantly and have been involved in the writing of the manuscript in draft and any revision stages, and have read and approved its final version.

## References

[1] Lepri G, Bellando-Randone  S, Distler JHW (2007-2015). Connective tissue diseases: Concept and pathogenesis, overlap syndromes, mixed CTD and undifferentiated CTD. EULAR on-line course on Rheumatic Diseases.

[2] Kamataki  A, Uzuki  M, Sawai  T (2015). Histopathological Change of Esophagus Related to Dysphagia in Mixed Connective Tissue Disease, Seminars in Dysphagia. InTech.

[3] Arbuckle  MR, McClain  MT, Rubertone  MV (2003). Development of autoantibodies before the clinical onset of systemic lupus erythematosus. N Engl J Med.

[4] Cervera  R, Khamashta  MA, Hughes  GR (1990). “Overlap” syndromes. Ann Rheum Dis.

[5] Kallenberg  CG (1995). Overlapping syndromes, undifferentiated connective tissue disease, and other fibrosing conditions. Curr Opin Rheumatol.

[6] LeRoy  EC, Maricq  HR, Kahaleh  MB (1980). Undifferentiated connective tissue syndromes. Arthritis Rheum.

[7] Reichlin  M (1976). Problems in differentiating SLE and mixed connective-tissue disease. N Engl J Med.

[8] Schneider HA, Yonker RA, Longley S, Katz P, Mathias J, Panush RS (1984). Scleroderma esophagus: a nonspecific entity. Ann Intern Med.

[9] Sharp GC, Irvin WS, Tan EM (1972). Mixed connective tissue disease—an apparently distinct rheumatic disease syndrome associated with a specific antibody to an extractable nuclear antigen (ENA). Am J Med.

[10] Marie  I, Dominique  S, Levesque  H, Ducrotte  P, Denis Hellot  MF (2001). Esophageal involvement and pulmonary manifestations in systemic sclerosis. Arthritis Rheum.

[11] Troshinsky  MB, Kane  GC, Varga  J, Cater  JR, Fish  JE, Jimenez  SA (1994). Pulmonary function and gastroesophageal reflux in systemic sclerosis. Ann Intern Med.

[12] Smolen  JS, Steiner  G (1998). Mixed connective tissue disease: to be or not to be?. Arthritis Rheum.

[13] Zuber-Jerger  I, Müller  A, Kullmann  F, Gelbmann  CM, Endlicher  E, Müller-Ladner  U (2010). Gastrointestinal manifestation of systemic sclerosis-thickening of the upper gastrointestinal wall detected by endoscopic ultrasound is a valid sign. Rheumatology.

[14] Sullivan WD, Hurst  DJ, Harmon  CE (1984). A prospective evaluation emphasizing pulmonary involvement in patients with mixed connective tissue disease. Medicine.

[15] Caleiro  MT, Lage  LV, Navarro-Rodriguez  T, Bresser A, da Costa  PA, Yoshinari  NH (2006). Radionuclide imaging for the assessment of esophageal motility disorders in mixed connective tissue disease patients: relation to pulmonary impairment. Dis Esophagus.

[16] Wigley  FM, Flavahan  NA (1996). Raynaud’s phenomenon. Rheum Dis Clin North Am.

[16.1] Knudson  RJ, Lebowitz  MD, Holberg  CJ, Burrows B (1983). Changes in the normal maximal expiratory flow-volume curve with growth and aging. Am Rev Respir Dis.

[17] Goldman  HI, Blecklake  MR (1959). Respiratory function tests: normal values at median altitudes and the prediction of normal results. Am Rev Tuberc.

[18] Gaensler  EA, Wright  GW (1966). Evaluation of respiratory impairment. Arch Environ Health.

[19] Bhalla  M, Silver  RM, Shepard  JO, McLoud  TC (1993). Chest CT in patients with scleroderma: prevalence of asymptomatic esophageal dilatation and mediastinal lymphadenopathy. AJR Am J Roentgenol.

[20] Wiener-Kronish  JP, Solinger  AM, Warnock  ML, Churg  A, Ordonez  N, Golden  JA (1981). Severe pulmonary involvement in mixed connective tissue disease. Am Rev Respir Dis.

[21] Kozuka  T, Johkoh  T, Honda  O, Mihara  N, Koyama  M, Tomiyama  N (2001). Pulmonary involvement in mixed connective tissue disease: high resolution CT findings in 41 patients. J Thorac Imaging.

[22] Saito  Y, Terada M, Takada  T, Ishida T, Moriyama  H, Ooi H (2002). Pulmonary involvement in mixed connective tissue disease: comparison with other collagen vascular diseases using high resolution CT. J Comput Assist Tomogr.

[23] Bodolay  E, Szekanecz  Z, De ´ve ´nyi  K, Galuska  L, Galuska  L, Csı ´po  I (2005). Evaluation of interstitial lung disease in mixed connective tissue disease (DMTC). Rheumatology.

